# Life‐threatening extrapleural hematoma in an anticoagulated patient 2 weeks after lobectomy

**DOI:** 10.1111/1759-7714.14160

**Published:** 2021-09-29

**Authors:** Masanori Shimomura, Masashi Iwasaki, Reona Shimegi, Masayoshi Inoue

**Affiliations:** ^1^ Division of Thoracic Surgery, Department of Surgery Kyoto Prefectural University of Medicine Kyoto Japan

**Keywords:** anticoagulant, chest wall, extrapleural hematoma, lung cancer, pleura

## Abstract

We report a case of an anticoagulated patient with sudden onset pleural hematoma after straining at defecation to cardiac arrest on 2 weeks after lobectomy for lung cancer. We decided to perform an emergent operation for an evacuation of extrapleural hematoma immediately after resuscitation. The bleeding point was revealed on the extrapleural thoracic apex. We should be aware that extrapleural hematoma can occur because of increased intrathoracic pressure such as straining during defecation in patients on anticoagulation.

## Graphical abstract

Extrapleural hematoma can occur because of increased intrathoracic pressure, such as that caused by straining during defecation in anticoagulated patients.

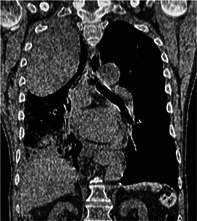

## INTRODUCTION

An extrapleural hematoma (EP) is a rare and occasionally life‐threatening condition defined as hemorrhage between the parietal pleura and endothoracic fascia. This condition may occur as a complication after blunt chest trauma associated with rib or sternal fractures, which cause injury to the intercostal or parasternal vessels and also be reported as a complication after thoracotomy to be treated conservatively.^1^ Here, we present a case of an anticoagulated patient with the sudden appearance of an EP 15 days after thoracoscopic lobectomy, requiring a surgical treatment because of a hemorrhagic shock.

## CASE REPORT

**FIGURE 1:**
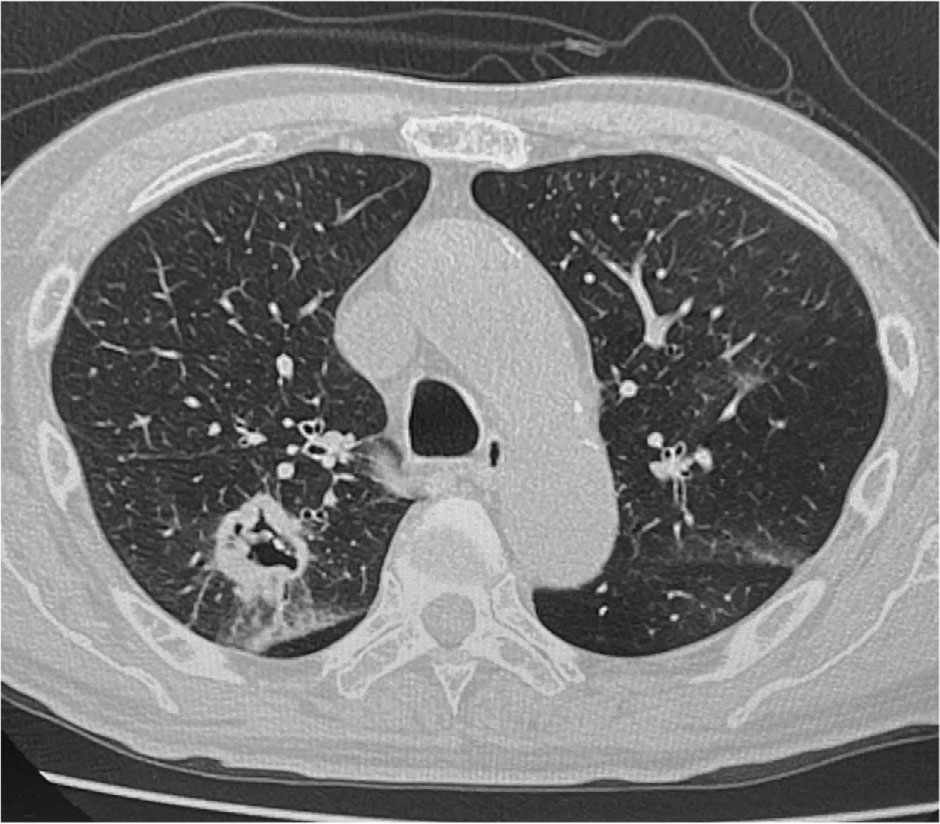
Chest computed tomography showing a 3.4 cm cavitary pulmonary nodule in the right upper lobe as clinical T2aN0M0 stage IB squamous cell carcinoma

**FIGURE 2:**
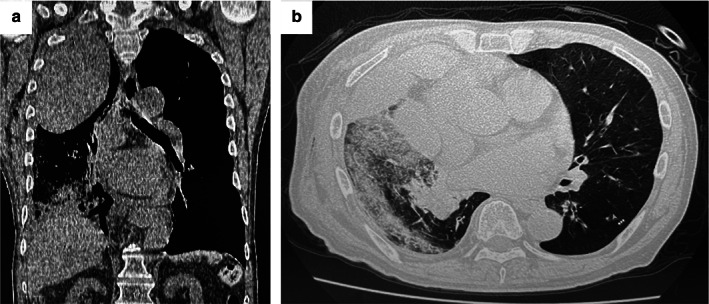
(a) Emergent chest computed tomography on 15 POD showed a huge extrapleural hematoma on the right anterior chest wall ranged to thoracic apex and (b) widespread ground glass opacification in the right lower lobe

An 86‐year‐old man was diagnosed with a squamous cell carcinoma of the lung after further examinations of hemosputum and was referred to our hospital for surgical treatment. A chest computed tomography (CT) scan showed a cavitary nodule without mediastinal nodal metastasis (Figure 1). The patient had a past history of brain infarction and atrial fibrillation 5 years previously, which was treated with medical treatment with apixaban. We replaced the anticoagulant drugs to intravenous heparin 7 days before his operation followed by a thoracoscopic right upper lobectomy and mediastinal lymph node dissection. The operation took 385 min because of intrathoracic adhesions and infiltrating hilar lymph nodes. We can detach the adhesion between parietal and visceral pleura except the part of the dorsal adhesion, in which the extrapleural detachment was required. The blood loss was 50 g. The pathologic staging was pT2aN0M0 stage IB. He progressed well after surgery, and the chest tube was removed on postoperative day (POD) 5. Regarding the administration of anticoagulant, we restarted the administration of intravenous heparin on POD 1 and switched it to apixaban on POD 5 (after removal of the chest tube). The chest X‐ray on POD 11 showed a small pleural space with effusion on the apex of the thoracic cavity. He suddenly developed a right chest pain and a hemoptysis after straining at defecation on POD 15. An emergent chest CT showed a huge extrapleural hematoma on the right anterior chest wall ranged to thoracic apex and widespread ground glass opacification in the right lower lobe (Figure 2). His vital signs were as follows: heart rate = 140 bpm, blood pressure = 85/52 mm Hg, SpO_2_ = 92% (nasal O_2_ 2L), and hemoglobin = 11.1 g/dl. The patient suddenly went into cardiac arrest probably because of a hemoptysis. We decided to perform an emergent operation for an evacuation of EP immediately after resuscitation. We evacuated the hematoma through 15‐cm incisions on the 4th and 7th intercostal space. The extrapleural space was occupied with a large amount of hematoma that ranged from thoracic apex to lower lateral thoracic wall. The bleeding point was revealed on the extrapleural thoracic apex after the removal of the hematoma to succeed in hemostasis by a suture (Video S1). The volume of bleeding was 840 g. No approach was made to the thoracic cavity because of the formation of postoperative adhesions in the thoracic cavity. Fortunately, the hemoptysis stopped under an overnight ventilator control, and we could remove the endobronchial tube on the next day. Extrapleural chest tubes were successfully removed 10 days after surgery. No evidence of EP was found for 6 months.

## DISCUSSION

EPs occur in ~7% of patients with blunt chest injuries.^2^ The extrapleural space is a rare site of blood pooling because of the limited space between the parietal pleura and thoracic muscle fascia. Usually, a watch‐and‐wait approach can be taken if the vital signs of the patient are stable and the hematoma does not expand in size. Several cases of conservative treatment for EP including observation or image‐guided drainage have been reported.^3^, ^4^ Although the residual EP often resolves spontaneously in the chronic phase after trauma, we have experienced cases in which the residual chronic EP produced pleural effusion that required surgical treatment.^5^ On the other hand, several cases of EP without episode of trauma were reported. Noguchi et al.^1^ reported EP as a complication 7 days after posterolateral thoracotomy for left lower lobectomy in an anticoagulated patient followed by a successful conservative therapy. Oka et al.^6^ reported that two patients with antiplatelet agents complained of pain in their shoulders or backs because of EP that originated from intercostal vessels.^6^ Sumida et al.^7^ also reported an anticoagulated case of EP followed by a successful evacuation of the hematoma.^7^ In this case, the patient had a right upper lobectomy that required total detachment of intrathoracic adhesions, and the residual space at the site of the apex of the chest cavity was prone to excessive traction on the parietal pleura. It was thought that the strong increase in intrathoracic pressure because of straining during defecation caused disruption of the subpleural blood vessels, resulting in an EP. Patients that were administered anticoagulant therapy could also have had possibilities of developing an appearance of rapid progression of EP. Major bleeding during treatment with apixaban is reported to be 2.13% per year.^8^ In this case, the cause of hemoptysis was still unknown, but postoperative condition with anticoagulant therapy had probably played some role in the hemoptysis. In conclusion, we should be aware that extrapleural hematoma can occur because of increased intrathoracic pressure such as straining during defecation.

## CONFLICT OF INTEREST

The authors declare that there is no conflict of interest for this work.

## Supporting information


**Video S1** Intraoperative finding after the evacuation of extrapleural hematoma. The bleeding point was revealed on the extrapleural thoracic apex.
